# Differential Thermal Stability and Oxidative Vulnerability of the Hemoglobin Variants, HbA_2_ and HbE

**DOI:** 10.1371/journal.pone.0081820

**Published:** 2013-11-14

**Authors:** Abhijit Chakrabarti, Dipankar Bhattacharya, Sanghamitra Deb, Madhumita Chakraborty

**Affiliations:** Biophysics and Structural Genomics Division, Saha Institute of Nuclear Physics, Bidhannagar, Kolkata, India; Indian Institute of Science, India

## Abstract

Apart from few early biophysical studies, the relative thermal instability of HbE has been only shown by clinical investigations. We have compared *in vitro* thermal stability of HbE with HbA_2_ and HbA using optical spectroscopy. From absorption measurements in the soret region, synchronous fluorescence spectroscopy and dynamic light scattering experiments, we have found thermal stability of the three hemoglobin variants following the order HbE<HbA<HbA_2_ in terms of structural unfolding and aggregation pattern. We have found formation of intermolecular dityrosine fluorophores with characteristic fluorescence signature, at pH >11.0 in all the three variants. Under oxidative stress conditions in presence of hydrogen peroxide, HbE has been found to be more vulnerable to aggregation compared to HbA and HbA_2_. Taken together, these studies have shown thermal and oxidative instability of HbE and points towards the role of HbE in the upregulation of redox regulators and chaperone proteins in erythrocyte proteome of patients suffering from HbEbeta thalassemia.

## Introduction

 Hemoglobin (Hb), the iron containing oxygen transport metalloprotein in red blood cells of vertebrates, is delicately structured and has highly fine-tuned, complex mechanism of action. Some abnormal forms of Hb or Hb variants, cause hemoglobinopathy, a genetic disorder that results in abnormal structure of the globin chain of Hb molecules e.g. sickle-cell anemia. Another hemoglobinapathy, thalassemia results from the reduced production of a specific type of globin chain(s) that otherwise are normal. Adult hemoglobin, HbA has two identical α chains each having 141 amino acid residues and two identical β chains, each having 146 amino acid residues and together form the tetrameric protein [1]. Approximately, one-fifth of the total surface area of the isolated subunits is buried in the process of forming the Hb tetramer [2]. Hemoglobin E (HbE; Glu26(B8)→Lys) is the most common Hb variant, prevalent in Southeast Asia [3]. The overall structure of HbE is similar to the structure of HbA. However, there are some local side chain alterations and the loss of the salt bridge interaction at the E26K_β_ mutation site. The reorientation of the side chains due to mutation, along with the changed network of water molecules leave HbE to be more unstable at high temperature [4-6]. HbA_2_, on the other hand have mutations at 10 different positions. Though none have an effect as dramatic as in the case of HbE, analysis from the x-ray crystallographic studies reveal that HbA_2_ has additional hydrogen bonds compared to HbA which play a positive role in the thermal stability of the variant over HbA and HbE [4]. 

 The structure, function and dynamics of hemoglobin have been studied for over a century leading to thousands of publications. However, solution studies involving conformational changes in Hb are not many. In the early 1970’s, following the X-ray crystallographic structure of hemoglobin and myoglobin by Max Perutz’s group, a series of conformational studies on Hb came up which were followed by a steady stream of work on structure, conformation and co-operative oxygen binding. Stability of hemoglobin in different pH and temperature has also been studied indicating complex unfolding behavior [7]. At a very low pH o f 1.8-3.0, heme dimerization takes place and Perutz showed that alkali has a milder effect on the denaturation of hemoglobin and 0.5M NaOH was required for a reversible dissociation of tetrameric hemoglobin into dimer [8]. Acid and alkaline unfolding and refolding of flounder Hb have been studied well in recent past. A molten globule like state below pH 2.5 has been identified with the maximum unfolding occurring at pH 2.5 [9,10]. Similar observations were made in trout hemoglobin where structural unfolding and oxidative property were found to be more pronounced and occurred at a faster rate in acidic pH (pH 1.5-3.5) than in alkali [11,12]. In unfolded state, with increasing structural melting, the heme group gets exposed to the environment resulting pro-oxidative activities in Hb where oxy-Hb (Hb-Fe^2+^--O_2_) or deoxy-Hb (Hb-Fe^2+^) first gets oxidized to met-hemoglobin (Hb-Fe^3+^) and then met-Hb, upon interaction with other reactive oxidative species (like H_2_O_2_) leads to formations of ferryl-Hb (Hb-Fe^4+^=O) and hemichrome causing oxidative damage to membrane lipids and proteins ultimately leading to hemolysis. These activities of hemoglobins, were also enhanced in acidic pH (pH 2.0-3.5) than in alkali, (pH 10.5-12.0) [9,11,13]. 

Studies on human Hb variants have been very few. Kinetics of thermal aggregation of HbC (β Glu6lys), HbS (β Glu6Val) and HbA_2_ at 45°C were followed by monitoring the change in the optical rotation at 233nm. Kinderlerer and coworker have shown that the three variants revealed a sigmoid type of thermal melting profile with inflexion points changing in the order HbS ^≈^HbC<HbA<HbA_2_. The time course for unfolding varied between the variants with HbS showing the lowest and HbA_2_ the highest half life [14,15]. The thermal denaturation studies gained further importance when it was observed that for many Hb variants, prolonged heating at temperatures over their inflexion points led to formation of irreversible aggregates in the form of hemichrome [16]. The molecular defect that gave rise to the instability of Hb variants was found to be responsible for hemichrome formation ultimately leading to hemolysis [17,18]. Drug induced oxidation of HbE was found to be faster than HbA, HbS and HbF [19]. It was recently shown that patients with HbE trait/HbH disease manifest severe anaemia during fever episodes [20]. Similar conclusions were drawn by Rees and Weatherall though their study involving the instability of HbE could not be correlated with the degree of thalassemia at physiological temperature [5]. 

These results have prompted us to further investigate the thermal denaturation properties of HbA_2_ and HbE. In the absence of any elaborate work on conformation and stability of HbA_2_ and HbE, we have taken up spectroscopic studies on thermal denaturation at different pH, purified from red blood cells of normal, β-carrier and homozygous HbE individuals respectively. We have used synchronous fluorescence and static as well as dynamic light scattering experiments to study the melting and aggregation behavior of these three hemoglobin variants at physiological as well as acidic and basic pH ranges. Erythrocytes are under oxidative stress during its life time. The major threat comes from hydrogen peroxide. Reactions of peroxides with heme proteins also results a covalent heme-protein cross-link. A new covalent heme-globin species has been identified and characterized [21-23]. Apart from membrane lipid peroxidation, the ferryl hemoglobin and free heme cause oxidative cross-linking of hemoglobin with membrane skeletal proteins spectrin to form high molecular weight aggregates in SDS-PAGE gels [24-27]. A recent structural & functional study on HbE has shown *in vitro* alteration of redox activity of HbE diminishing nitrated reducing ability by 2.5 times and increasing redox potential of HbE from HbA [28]. This study has also shown reduced number of hydrogen bonds in the microenvironment of β26Lys. Our work on proteomics studies have indicated up-regulation of certain redox regulating proteins and enhanced levels of reactive oxygen species (ROS) in HbEβ-thalassemia. We have also found out strong correlation of levels of those proteins with HbE levels in the patients [29,30]. These results also prompted us to further study the oxidative stability of HbE with respect to HbA and HbA_2_. 

## Materials and Methods

### Materials

Blood samples, collected in EDTA vials (BD Biosciences), from normal healthy volunteers and homozygous HbE individuals, were used for the purification of HbA, HbA_2_ and HbE respectively, elaborated in our earlier work [4]. Blood samples from normal healthy volunteers and homozygous HbE individuals were obtained from Ramakrishna Mission Seva Pratishthan, with informed written consent following the guidelines of the Institutional Ethical Committee of Vivekananda Institute of Medical Sciences, Ramakrishna Mission Seva Pratishthan, Kolkata 70026, INDIA and Institutional Animal & Bio ethics committee of Saha Institute of Nuclear Physics. The Institutional Ethical Committee of Vivekananda Institute of Medical Sciences, Ramakrishna Mission Seva Pratishthan, and Institutional Animal & Bio ethics committee of Saha Institute of Nuclear Physics have also specifically approved the current study. Percoll, SP-Sephadex, Tris-HCl, sodium citrate, sodium acetate, MES, CAPS and acrylamide were purchased from Sigma. Sodium monohydrogen phosphate, sodium dihydrogen phosphate and glycene were purchased from Promega (Madison, WI). Amicon Ultra™ spin columns were purchased from Millipore (Bedford, MA). All cuvettes were purchased from Hellma GmBH (Müllheim, Germany). Other chemicals, if not otherwise mentioned, were purchased locally and were of spectroscopy grade. 

### Purification of Hemoglobin variants

Erythrocytes were purified from blood samples using 75% percoll gradiant and hypotonic lysis of purified erythrocytes was done as described in our earlier work [4,31]. HbA and HbA_2_ were separated by ion-exchange chromatography on SP-Sephadex column, from normal hemolysate and HbE was purified from homozygous HbE (HbEE) hemolysate [31] with minor modification of our previous work [32]. HbA and HbA_2_ (or HbE) were separated at an elution rate of 18 ml/hour using increasing salt gradient of the buffer containing 10 mM phosphate, pH 6.7, 0.16 M NaCl and 0.01% (w/v) KCN using the gradient mixer pump in the Biologic™ FPLC system of BioRad. HbA was eluted at lower salt gradient, at ~0.01-0.015 M NaCl, followed by HbA_2_ (or HbE) at 0.1-0.12 M NaCl. The purified hemoglobin variants were dialyzed against a buffer containing 10 mM Tris-HCl, pH 10 with 0.1 M NaCl and were concentrated to ~ 50 mg/ml (~0.8 mM) and stored in aliquots of 100 µl at -80°C for not more than two weeks. Protein concentration of each variant was measured using both the Lowry’s method of protein estimation and by measuring the Soret absortion at 415 nm using molar absorption coefficient (**ε**) of 125000 M^-1^. 

### UV-Visible Absorption Studies

The pH induced unfolding studies were carried out in a Unicam UV-500 (Thermospectronic, NY) dual beam spectrophotometer coupled with a heating peltier bath. Prior to each set of experiment, the concentration of hemoglobin stock was measured and for absorption studies it was taken to be 10µM at pH 7.0. The absorption spectra were recorded in range from 250nm-700 nm. Hemoglobin exhibits four distinct absorption peaks at 345 nm, 415 nm, 541 nm and 577 nm respectively in its oxy form [33]. Multiple scans were taken and was averaged to record the final scan. The background subtraction for buffer was done in the instrument itself before saving the spectra. The saved final spectra were analyzed using Microcal ORIGIN™ (V5.0) software package. 

### Synchronous Fluorescence Spectroscopic Studies of Hemoglobin Variants

Synchronous fluorescence spectra of hemoglobin have been recorded in FluoroMax-3 spectrometer (Horiba Jobin Yvon, IBH) and analyzed to study changes around tryptophan and tyrosine residues as a function of temperature for HbA, HbA_2_ and HbE. After incubating 1µM protein for 30 minutes at 25°C, synchronous fluorescence spectra were recorded at intervals of Δλ= 20 nm, corresponding to tyrosine and at intervals of Δλ = 80 nm, corresponding to that of tryptophan [34]. The spectrum was scanned from 200nm-500 nm with a scan speed of 1nm/sec. The final spectrum was averaged from three scans. The resulting spectra, after subtracting contributions of the buffer, were analyzed using Origin 5.0 software package. 

### Dityrosine Fluorescence in Hemoglobin Variants

The formation of DT in Hb variants was monitored in alkaline pH from the fluorescence signal of the DT fluorophore at 400nm while exciting it at 315nm. The temperature scans for this newly generated DT fluorophore were also recorded with excitation at 315nm. 

### Thermal Denaturation Studies

Preliminary study on Hb denaturation as a function of pH ranging from 1 to 11.5 indicated that unfolding started from pH 4 and below pH 2.5, it showed complete unfolding. We have chosen pH 2.5, 4.0, 7.0 and 11.5 for thermal denaturation studies using uv-visible absorption and synchronous fluorescence spectroscopic techniques. Hb concentrations for fluorescence studies were kept 1µM and that of absorbance studies was 5 µM respectively. For all experiments, the respective Hb variant was incubated in the corresponding buffer at 4°C for 30 minutes. For each temperature, the protein solution was incubated for at least 5 minutes after attaining the desired temperature. The slit width for synchronous fluorescence was kept at 5nm each for excitation and emission wavelength. The thermal unfolding studies were carried out in a range from 10°C to 65°C with increments of 5°C intervals. However, after analysis of initial experimental data, the increment of temperature near the inflexion region (i.e. 33°C-50°C) was done at an interval of 3°C. Thermal unfolding studies were carried out at pH 2.5, 4.0, 7.0 and 11.5 in buffers of Glycine-HCl, Acetic acid/Sodium acetate, Tris-HCl and CAPS/NaOH respectively. 

### Thermal Aggregation Studies

To investigate the relative thermal stabilities of the hemoglobin variants, static and dynamic thermal aggregation studies were carried out at a particular pH. HbE (45 µM) was taken for static thermal aggregation studies. 90° light scattering intensities were recorded at 500 nm in fluorescence spectrometer. The recorded data was analyzed with Origin 5.0 software package. 

A temperature controlled DynaPro MS 800 (Wyatt Technologies, Santa Barbara, CA) dynamic light scattering spectrometer was used to measure individual steps of the temperature controlled unfolding and aggregation of the three hemoglobin variants. Prior to measurement, all buffer samples were filtered repeatedly with the 0.02 µm Anodisc™ membrane filter with a stainless steel filter assembly provided by the manufacturer. The protein solutions were added to the filtered buffers at a final concentration of 3 mg/ml (~46 µM) and were re-filtered using Anodisc™ filter of 0.1 µm to ensure minimized contamination from environment. 50 µl of the filtered sample was taken into a specially fabricated quartz micropipette and were incubated in increasing temperatures from 10°C to 70°C. The hydrodynamic radius of the sample, primarily measured in DLS, has been calculated on the basis of a reference protein BSA. The correlation between the calculated and actual data were reflected by the SOS values, the lower value of which implied better homogeneity of the solution in terms of particle sizes. For our calculations, data points with SOS values >100 were discarded and the solutions with high initial values were re filtered. The resultant data points were exported and analyzed. 

### Studies on Thermal Stability under Oxidative Conditions

To study the oxidative vulnerability of the hemoglobin variants, 45 µM of each hemoglobin variant was taken separately in 20 mM filtered phosphate buffer, pH 7.4. H_2_O_2_ (5 mM) was added into the protein solution and the kinetics of aggregation in terms of change in hydrodynamic radius was studies using the DynaPro DLS instrument. The time dependent changes in the scattering intensity data was averaged over every 5 seconds interval and the resultant plots of time versus radius for HbA, HbA_2_ and HbE were processed using Origin 5 software package. 

To ascertain the effects of H_2_O_2_ on the structural integrity of the three hemoglobin variants, 20 µg of HbA, HbA_2_ and HbE were treated with 5 mM of H_2_O_2_ in 20 mM phosphate buffer for 30 minutes. After the incubation, each hemoglobin variant was boiled with SDS-PAGE running buffer and was run in a 11 cm, 4-20 % criterion gel (BioRad) and the band intensities of different hemoglobin variants were noted. Prior to running of the gel, the proteins were incubated in 50 mM DTT for 30 minutes to cleave the disulphide bonds. 

## Results

### Thermal Unfolding of HbA, HbA_2_ and HbE at different pH

The thermal melting studies of the three hemoglobin variants were carried out using both uv-visible absorption and synchronous fluorescence spectroscopic methods. To have a closer look at the changes around the tyrosine and tryptophan residues exclusively, synchronous fluorescence experiment was performed where the scanning of wavelengths was done by keeping the wavelength difference between excitation and emission, Δλ, at 20 nm for the tyrosine residues and using the Δλ value at 80 nm, for the tryptophan residues. In the absorption measurements, the main information was obtained by tracking the changes in absorbance and the peak shift in the Soret region indicating the changes occurring near the heme environment. 

**Figure 1:**
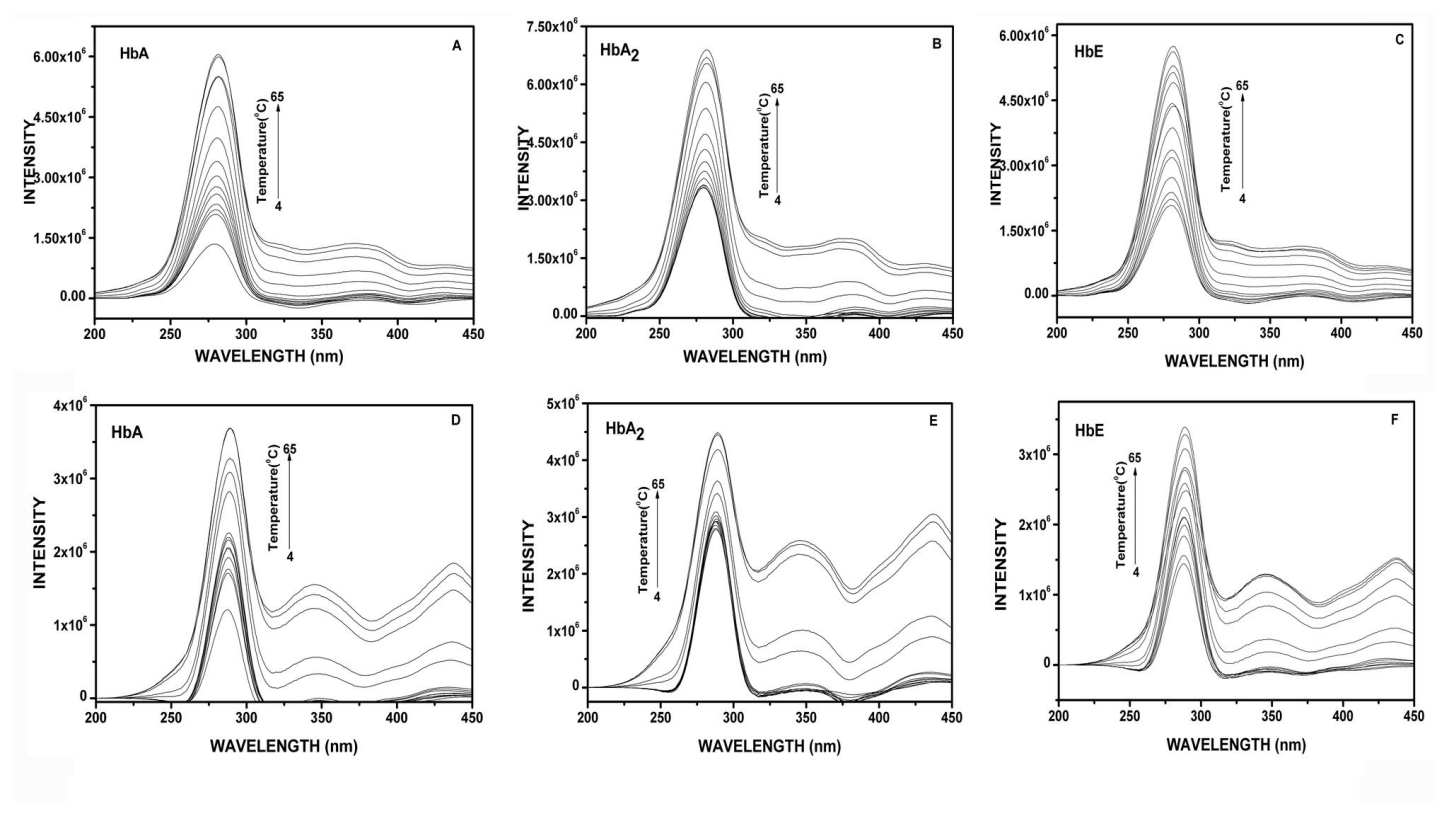
Synchronous fluorescence spectra for the hemoglobin variants (1.0 µM) HbA, HbA_2_ and HbE with increasing temperature from 4°C-65°C. (**A**-**C**) Represent the spectra for tryptophan (∆λ=80 nm) with main peak at around 280nm and auxiliary peak at 374nm and (**D**-**F**) represent those for the tyrosines (∆λ=20 nnm) with main peak at around 288nm and two auxiliary peaks at 348nm and 435nm. All spectra were taken at pH 7.0. The increasing intensities denote the solvent exposure of tyrosine and tryptophan residues. Auxilliary peaks in tryptophan indicate generation of other fluorophores or tyrosine derivatives.

**Figure 2:**
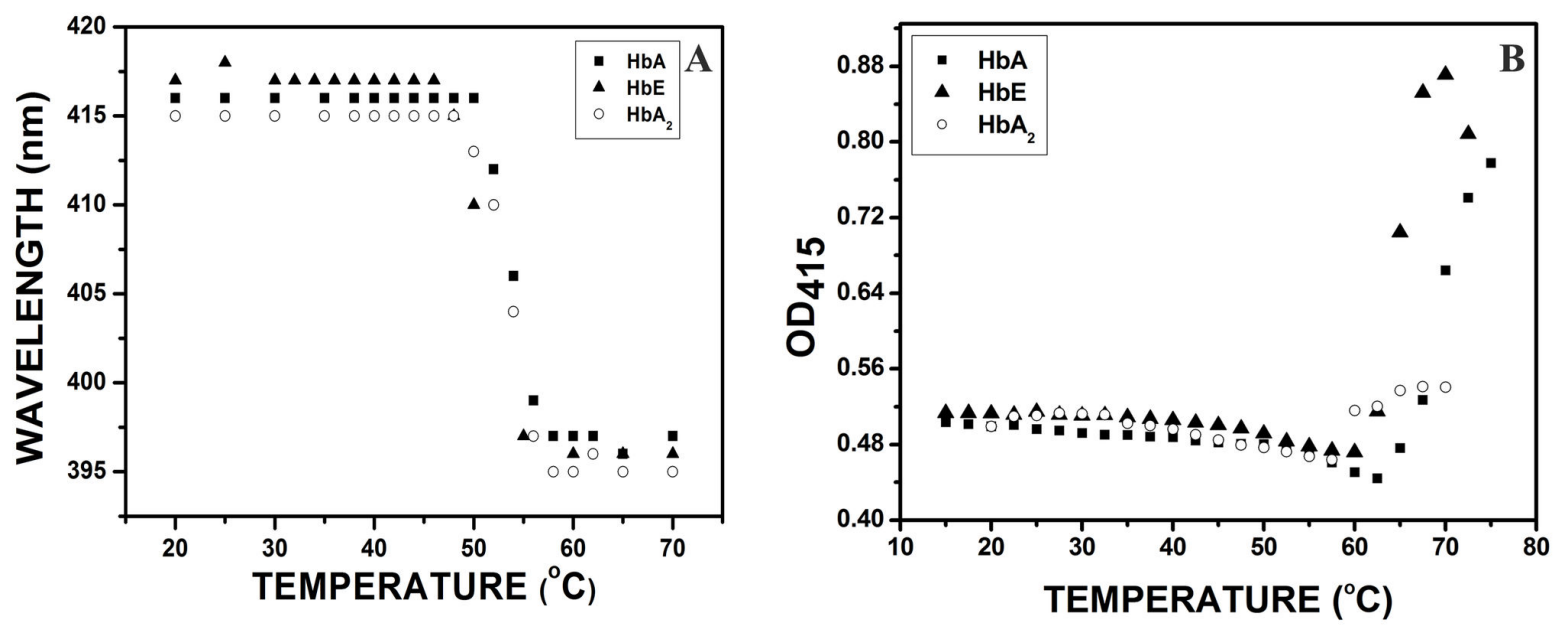
Thermal unfolding of HbA, HbE and HbA_2_ (10.0 µM) at pH 7.0 as recorded from (A) the change in the wavelength of Soret absorption maxima (415nm to 395nm) with increasing temperature, where bathochromic shift observed at temperature above 50°C, and (B) the change in absorbance in the Soret region (415nm) measured by uv-visible absorption spectroscopy. The increased intensities beyond 60°C is due to aggregation of globin chains at higher temperature.

In fluorescence studies when the three variants were heated from 4°C to 65°C at pH 7, the results obtained from synchronous fluorescence are given in Figure **1**. Figure **1 A-C** represents the synchronous fluorescence spectra for tryptophan for HbA, HbA_2_ and HbE respectively with the main peak at ~ 280 nm and auxiliary peak at 374 nm. With increasing temperature, the intensity increases steadily with a minor bathochromic shift of 2 nm at 65°C for both the peaks. However, for the tyrosine spectra (Figures **1 D-F**), apart from the main peak at 288 nm, two more auxiliary peaks at 348 nm and 435 nm appear. The intensities of all the three peaks increased with increasing temperature, indicating solvent exposure of the fluorophores as a result of unfolding. The increased intensities of the auxiliary peaks at 348 nm and 435 nm are probably indicative of generation of new fluorophores, excitable at 280 nm or formation of certain tyrosine derivatives. Analysis of the above data indicate the inflexion temperatures for HbA, HbA_2_ and HbE, obtained from the plot of changes in synchronous fluorescence intensity of tryptophan and tyrosine as a function of temperature and from the X_Y50_ values from the linear portions of the sigmoidal fit to the data points. The results are summarized in Table **1**. The uv-visible absorption studies at pH 7.0, also shows the unfolding of the hemoglobin variants with respect to temperature like those obtained from the fluorescence studies. The state of unfolding, evident from the increase in Soret absortion at 415 nm and hypsochromic shift of the absorption maxima from 415 nm to ~395 nm was not as sharp as obtained from fluorescence studies, mainly because of thermal aggregation of the unfolded globin chains that contributed in the increase in absorbance as well. The results are shown in Figure **2**. The relatively higher transition temperatures obtained from the absorption studies are indicative of the fact that during thermal unfolding, the changes occurring around heme starts at a much higher temperature than the other parts of the globin chains. 

**Table 1:** Inflexion points from the temperature dependent synchronous fluorescence studies for HbA, HbA_2_ and HbE at pH 7.

**Hb Variant**	**Synchronous Fluorescence (Tyrosine) (Δλ = 20 nm) (°C)**	**Synchronous Fluorescence (Tryptophan) (Δλ = 80 nm) (°C)**
HbA	41.2±3.1	45.2
HbA_2_	45.9±1.0	51.2±1.1
HbE	37.7±1.9	42.9±3.4

The error bars are Standard Errors of Mean (SEM) of 5 independent experiments.

**Figure 3:**
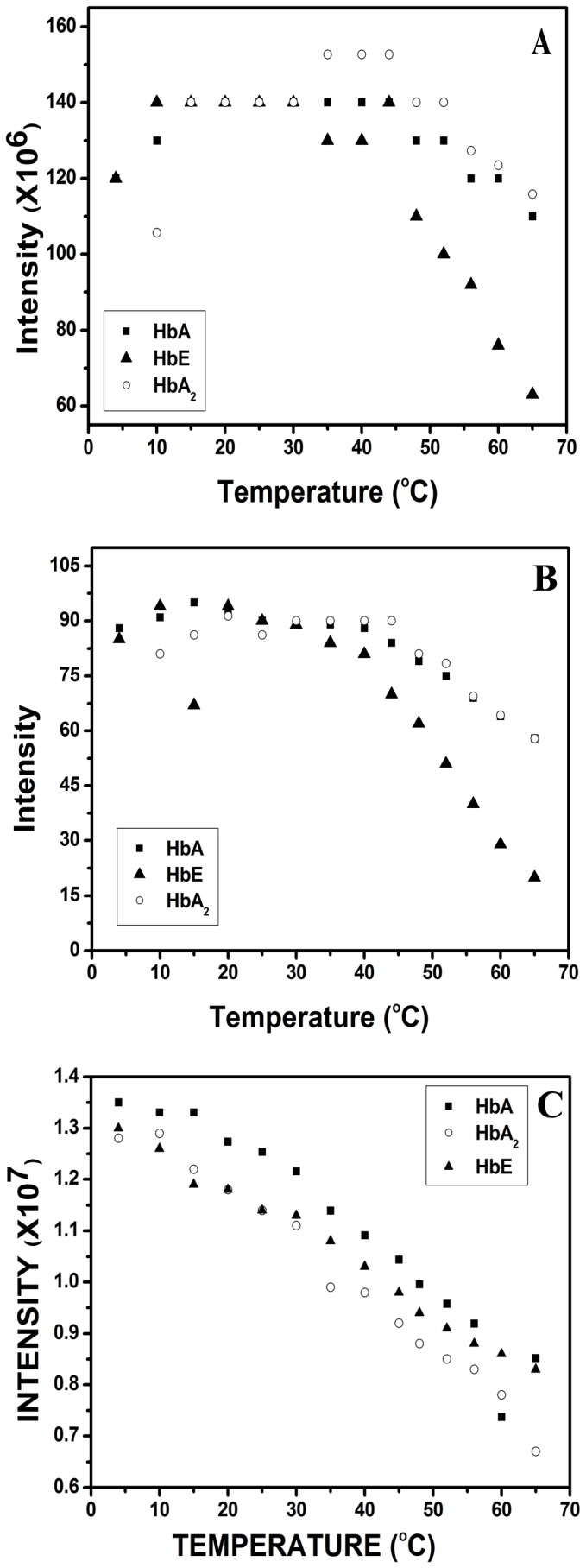
Change in fluorescence intensity in the HbA, HbA_2_ and HbE (5.0 µM) as a function of temperature at pH 4.0. The changes are reflected by (**A**) synchronous fluorescence for tryptophan emission, (**B**) synchronous fluorescence for tyrosine and (**C**) Change in synchronous fluorescence for tryptophan of HbA, HbA_2_ and HbE (1.0 µM) as a function of temperature at pH 2.5. Sharp decrease in intensity values in (A) and (B) indicate formation of unstable and insoluble aggregates which starts at a much lower temperature for HbE than HbA & HbA_2_ while at pH 2.5 all the variants become much more temperature sensitive as they are completely unfolded.

 The pH induced unfolding studies of hemoglobin variants revealed that unfolding occurs via a two step transition, the first one around pH 5-6 and another around pH 2.5 at which all the three hemoglobin variants were maximally unfolded. This prompted us to study the unfolding behavior of HbA, HbA_2_ and HbE at pH other than the physiological range, especially in the acidic regions of pH 2.5 and pH 4.0. On lowering the pH to 4.0, intensities for the synchronous emission for tryptophan and tyrosine increases upto 25°C following which the intensity values remain more or less static upto 35°C. Beyond this point, the intensity for HbE starts decreasing sharply with rise in temperature while that for HbA and HbA_2_ remains more or less constant till ~ 50°C, shown in Figure **3A, 3B**. The probable reason for the decrease might be the formation of insoluble aggregates resulting in a reduction in the concentration of proteins. For HbE, the fall in the intensity is much sharper and initiated at a temperature between 35-40°C. At a further lower pH of 2.5, all the hemoglobin variants are completely unfolded. At pH 2.5 the emission maxima appeared at 352 nm which on further heating slowly shifted to 356 nm and the fluorescence intensities steadily decreased (not shown). It was observed that at lower pH values, the synchronous fluorescence maxima did not shift appreciably and showed one sharp peak for both tryptophan and tyrosine. Figure **3C** represents the change in synchronous fluorescence intensities for tryptophan of the three Hb variants with respect to temperature. 

**Figure 4:**
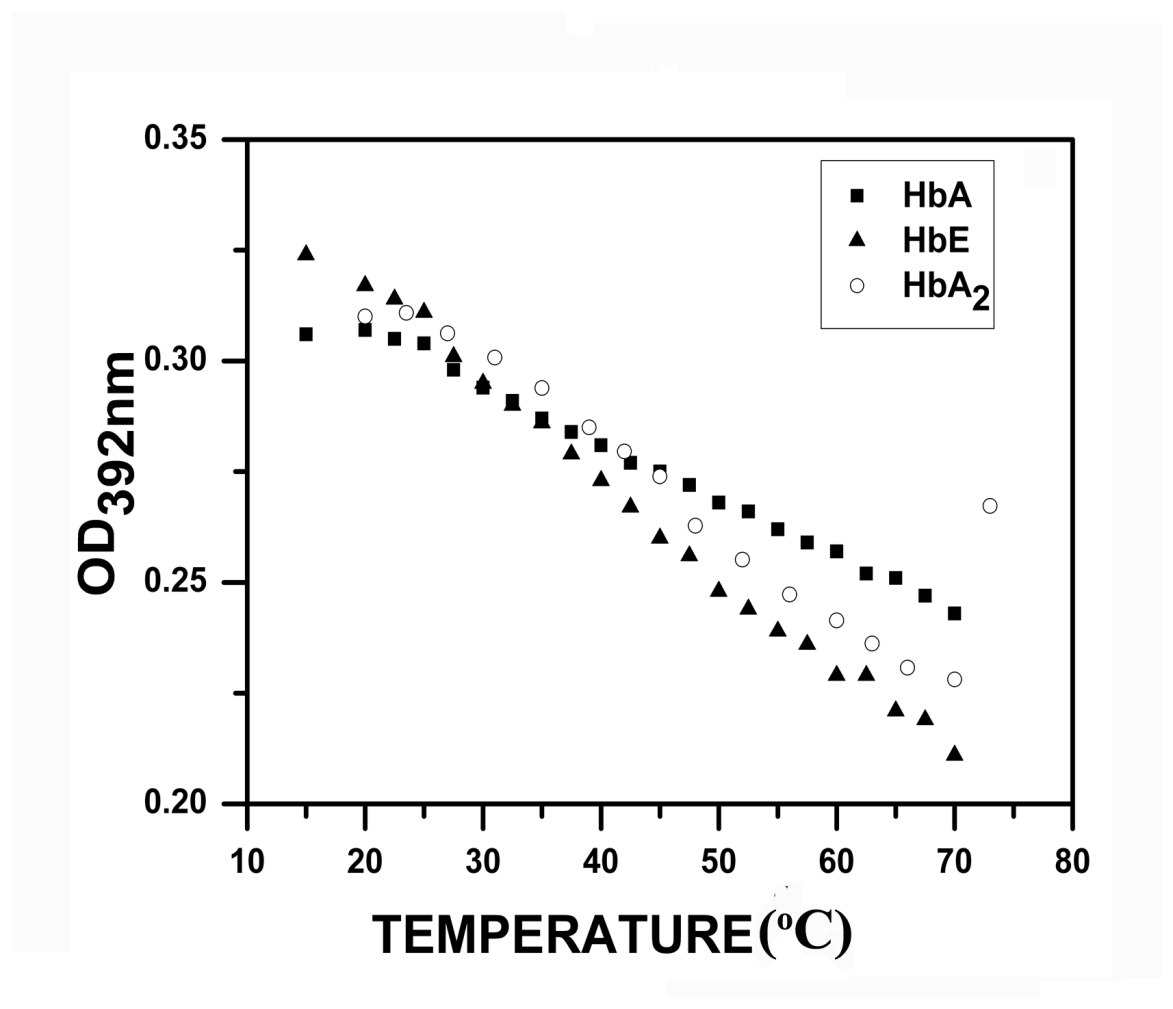
Change in absorbance in HbA, HbA_2_ and HbE (10.0 µM) as a function of temperature at pH 2.5. The slope of the lines indicate the order of thermal instability to be HbA>HbA_2_>HbE at pH 2.5. The error bars are Standard Errors of Mean (SEM) of 5 independent experiments which are of the order of the size of the symbols.

It is also evident from the uv-visible Soret absorption spectra of the hemoglobin variants where the normal characteristic spectra of hemoglobin with main Soret absorption peak at 415-416 nm gives way for a broad uncharacteristic peak at ~ 392 nm which on heating shows a gradual red shift to ~ 397 nm at pH 2.5 (not shown ). Origin of this unusual peak shift may result due to the degradation of the heme-globin associated complex resulting in the generation of free heme from the unfolded and aggregated globin chains with emission at 406 nm. The plot of OD_392_ against temperature at pH 2.5 also showed the same pattern as obtained from the fluorescence studies, shown in Figure **4**. The slope of the straight line decreases in the order HbA>HbA_2_>HbE, indicating that at pH 2.5, HbA has higher thermal stability than HbA_2_. 

### Thermal Denaturation at pH 11.5: Formation of Dityrosine

**Figure 5:**
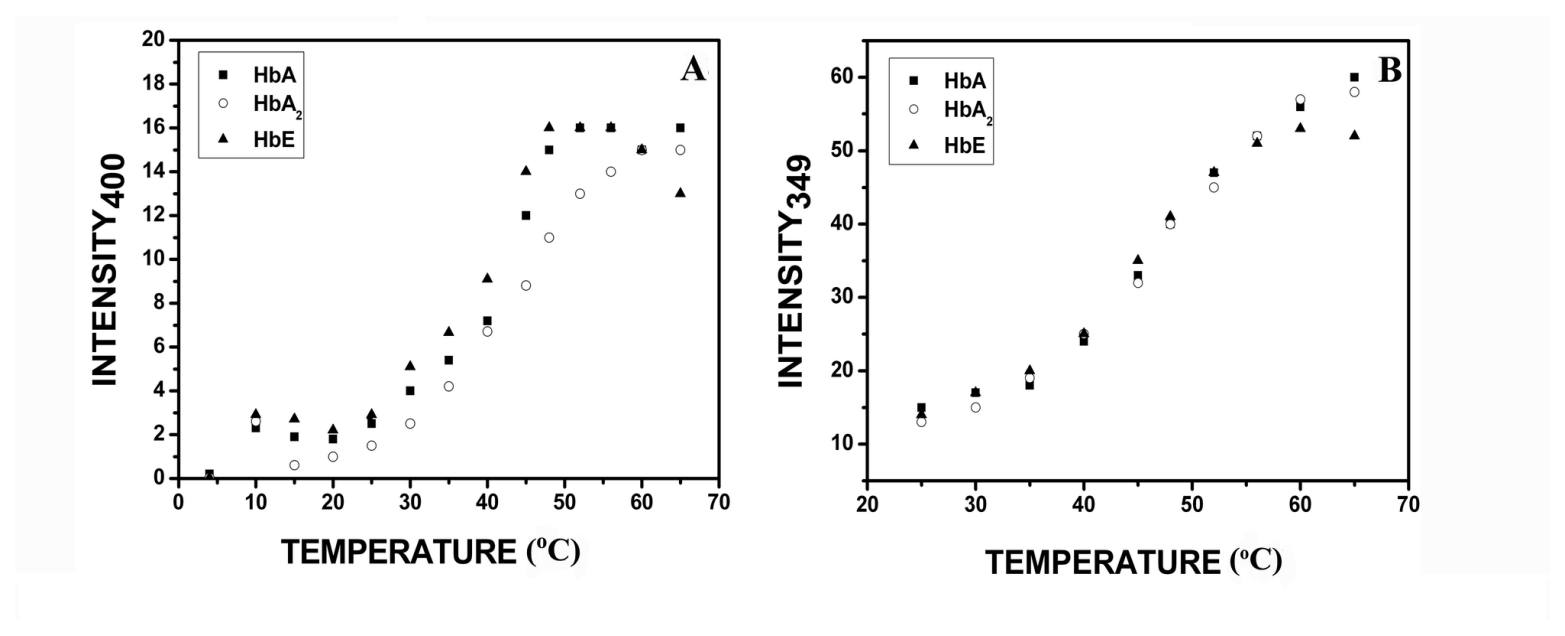
Changes in intensities of (A) for dityrosine (Ex 315 nm, Em 400 nm) formed and (B) synchronous fluorescence of tyrosine in HbA, HbA_2_ and HbE (5.0 µM) as a function of temperature at pH 11.5. The complete scan for the spectra related to (**A**) and (**B**) are presented in Figures S1 and S2 respectively.

The effect of pH on the folding of hemoglobin variants were much less penetrating in alkaline range than acidic range as we have observed it from the pH induced unfolding studies, also known in literature [11]. The most remarkable difference is observed from the tyrosine emission and the synchronous fluorescence spectra of all the three variants at pH 11.5. It was observed that with rise in temperature an auxiliary peak starts appearing at 400 nm in the tyrosine synchronous fluorescence. After a certain temperature, the auxiliary peaks become the major peak. It was obvious that at pH 11.0 and beyond, a new fluorophore was generated at elevated temperature which was completely absent in acidic pH range, characterized to be dityrosine (DT) with excitation and emission maxima of 315 nm and 400 nm respectively [35-37]. Thus the temperature scans for this newly generated DT fluorophore were also recorded with excitation at 315nm. The results are shown in Figure **S1**. The tyrosine synchronous fluorescence spectra for HbA, HbE and HbA_2_ showing the generation of new fluorophore with rising temperature at pH 11.5 is shown in Figure **S2**. Reducing agents like DTT and glutathione inhibits DT formation [38] and that was also verified experimentally by heating HbA at 50°C in buffer of pH 11.0 in presence of 100 mM DTT where the emission intensity was reduced substantially which confirms the formation of DT, also found in our earlier work with RFX proteins [39]. The analysis for temperature variation of DT formation in HbA, HbA_2_ and HbE and the tyrosine synchronous fluorescence of these three variants are shown in Figure **5A & 5B** and results are summarized in Table **2**. It is also noteworthy that even at alkaline pH, the relative thermal stability between HbA, HbA_2_ and HbE is conserved as is seen in neutral to physiological pH range. This also emphasizes the fact that HbA_2_ maintains its conformation which is responsible for its high thermal stability even when it is partially unfolded. Only on complete unfolding (pH 4.0 and below) HbA_2_ loses its advantage, and at highly acidic pH ranges, HbA becomes thermally more stable. 

**Table 2:** Inflexion points from the temperature dependent Dityrosine and synchronous fluorescence measurements for HbA, HbA_2_ and HbE at pH 11.5.

**Hb Variant**	**I_400_ (Dityrosine)(°C)**	**Synchronous Fluorescence** **for Tryptophan (°C)**	**Synchronous Fluorescence for Tyrosine (°C)**
HbA	41.5±0.9	38.8±0.9	47.2±0.4
HbA_2_	43.3±0.1	40.8±1.0	46.8±0.6
HbE	38.7±1.5	38.2±0.7	46.6±0.6

The error bars are Standard Errors of Mean (SEM) of 5 independent experiments.

**Figure 6:**
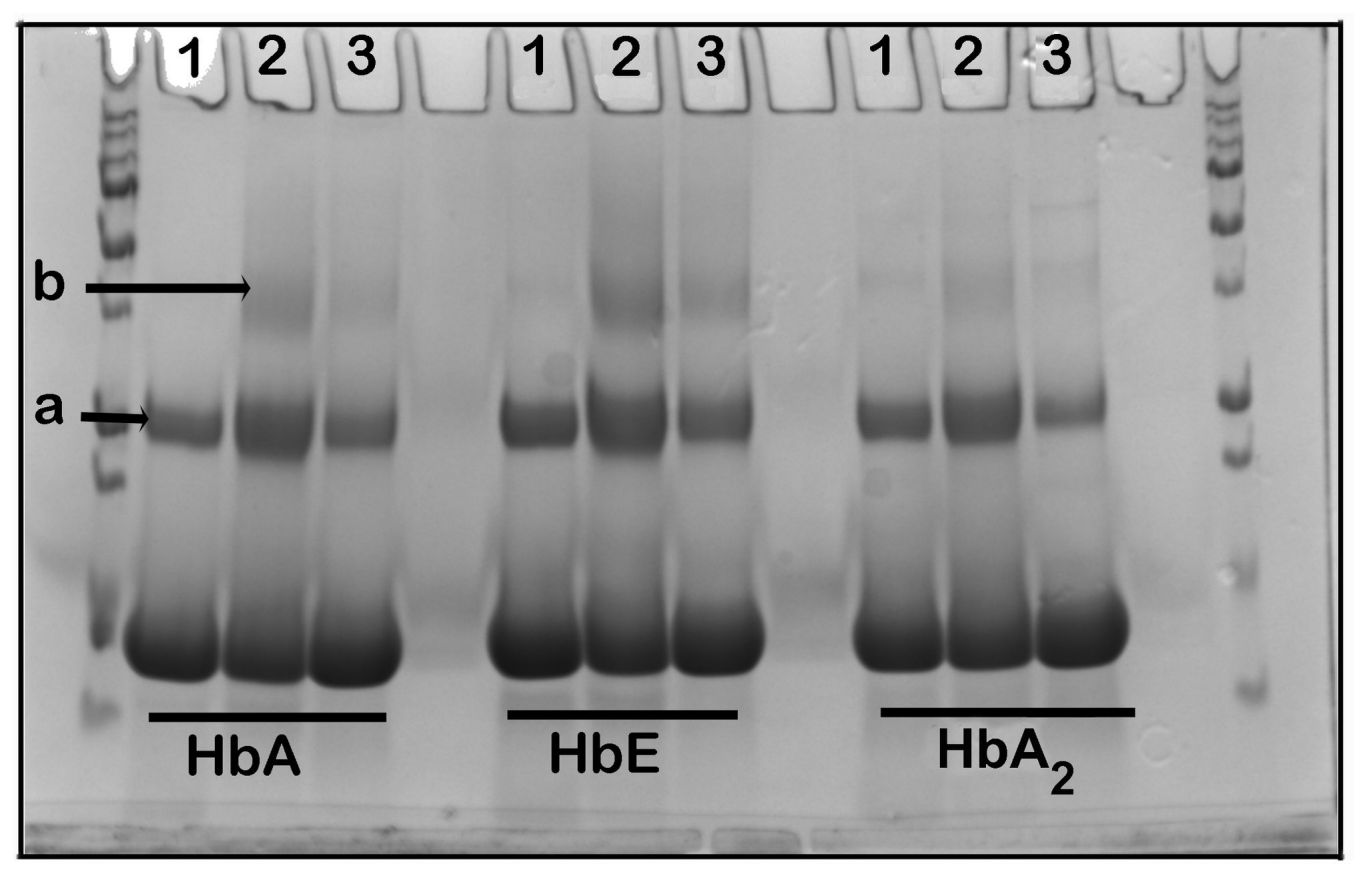
SDS-PAGE analysis of Hb variants (20 µg) incubated at pH 11.5 and at 40°C for 15 minutes. Lane 1 shows the control, untreated Hb. Lane 2 shows the same after incubating in 5 mM H_2_O_2_ at pH 11.5 for 30 minutes and Lane 3 shows the same, as in Lane 2, after further incubation in 10 mM DTT for 15 minutes showing maximum yield of high molecular weight aggregates (**band** **a** and **band** **b**) in HbE than HbA and HbA_2_. Also appearance of band b in presence of H_2_O_2_ (Lane 2) and their disappearance in presence of DTT (Lane 3) indicate formation of intermolecular dityrosine.

To check whether DT is intermolecular or intramolecular in nature, the Hb variants, incubated in buffer of pH 11.5 at 40°C for 15 minutes, were run in SDS-PAGE at high concentration in presence and absence of 5 mM H_2_O_2_. The results are shown in Figure **6**, indicating formation of high molecular weight species (band ‘a’) in control lanes (protein in pH 11.5 buffer) and a higher molecular weight species (band ‘b’) in presence of H_2_O_2_. The band ‘b’ disappeared when the protein was incubated with H_2_O_2_ in presence of 10 mM DTT. Also, in presence of DTT, density of band ‘a’ was reduced. The densitometric analysis of the higher molecular weight bands clearly showed that the yield of such species is higher in HbE than HbA and HbA_2._

### Thermal Aggregation of HbA, HbA_2_ and HbE

**Figure 7:**
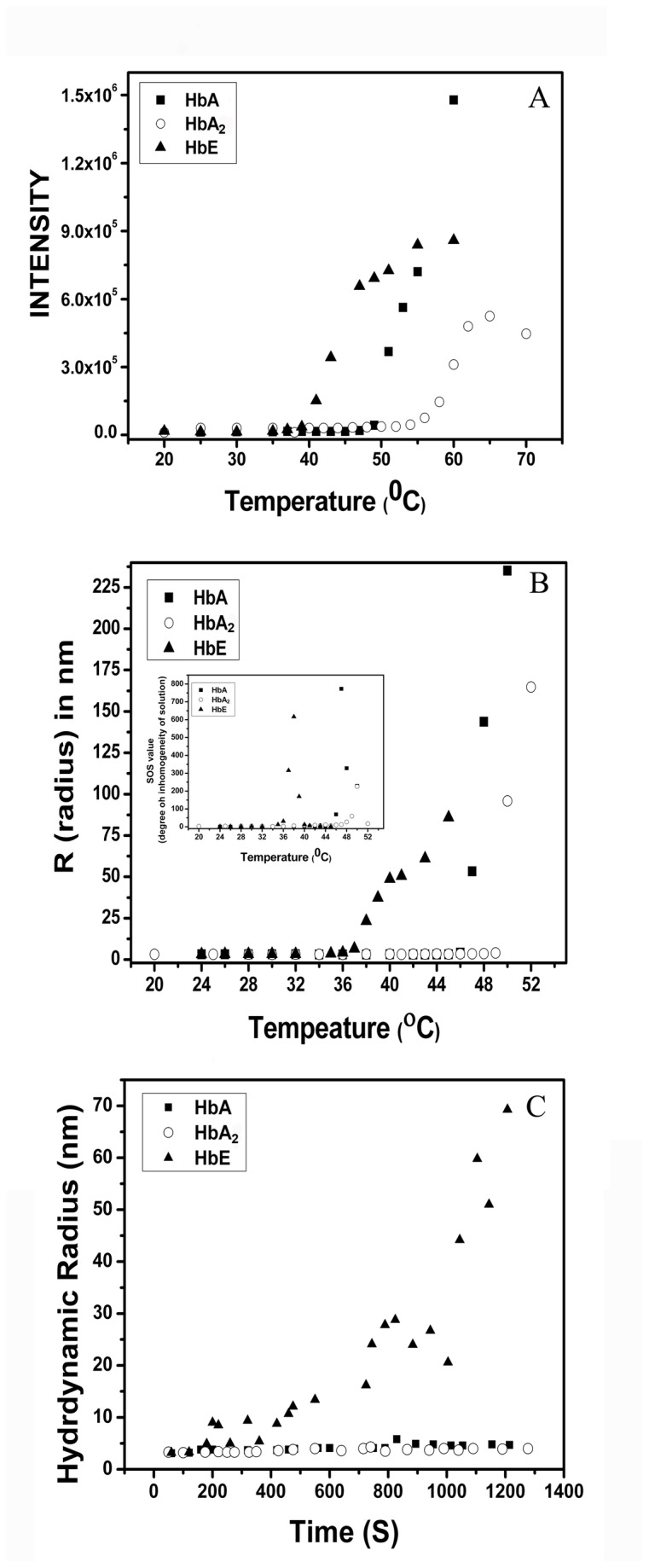
Thermal aggregation studies of HbA, HbA_2_ and HbE (45.0 µM) in presence and absence of 5 mM H_2_O_2_. (**A**) Change in the intensity of 90° light scattering at 500 nm measured in a fluorescence spectrometer at pH 7 and (**B**) the same in the hydrodynamic radius as calculated by DLS measurements. Inset of (**B**) represents the homogeneity of the species under the light scattering experimental condition in terms of SOS values. Higher value indicates more heterogeneity in the solution in terms of radii of the species present. (**C**) Oxidative instability of HbA, HbA_2_ and HbE (45.0 µM) in presence of 5 mM H_2_O_2_ as reflected in the kinetics of aggregation, obtained from the change in hydrodynamic radius of the Hb variants with time. HbE shows a marked instability compared to the rest of HbA and HbA_2_ where aggregation starts within 180 second of monitoring.

It was observed from the thermal unfolding experiments that all the three variants have shown differential thermal unfolding pattern over a wide pH range starting from pH 2.5 to pH 11.5. It was also observed that in almost all experiments, the fluorescence intensity tends to drop after attainment of a particular temperature which was also pH dependent. One probable reason for decrease in fluorescence was aggregation of the respective hemoglobin variant. To test the hypothesis, all three variants were subjected to aggregation studies using 90° light scattering measurements as well as dynamic light scattering at the most stabilizing pH 7.0, shown in Figure **7**. The results clearly show a distinct difference in the temperature at the onset of aggregation from 90° light scattering and dynamic light scattering data. Both the experiments showed HbE to be much more susceptible to agregation than HbA and HbA_2_ while between them, HbA_2_ is more stable towards thermal aggregation. From the Boltzmann analysis of the results, the inflexion temperatures were obtained and have been tabulated in Table **3**. 

**Table 3:** Inflexion points from the temperature dependent light scattering measurements for HbA, HbA_2_ and HbE.

**Hb Variant**	**90° Light Scattering (°C**)	**DLS (°C)**
HbA	55.6±0.9	47.7±0.06
HbA_2_	59.3±0.2	49.9±0.1
HbE	44.2±0.4	39.8±0.5

The error bars are Standard Errors of Mean (SEM) of 5 independent experiments.

### Oxidative Vulnerability of HbA, HbA_2_ and HbE

The formations of DT and high molecular weight aggregates were more facile in HbE at alkaline pH than HbA and HbA_2_. It has also become important physiologically after the identification of more abundance of redox regulator proteins in HbEβ-thalassemic erythrocytes [29]. These two observations led us to check for the oxidative stability of the three hemoglobin variants with respect to their aggregation kinetics under oxidative stress in presence of 5 mM H_2_O_2_ at 20°C and mimics *in vitro* the conditions inside erythrocyte where superoxide and other reactive oxygen species are converted into H_2_O_2_ by the action of catalase and superoxide dismutase. The change in hydrodynamic radius of the particular hemoglobin variant was monitored with respect to time by DLS measurements, shown in Figure **7C**. HbE started aggregating almost immediately upon incubation with H_2_O_2,_ and with time the aggregates started growing in size while HbA and HbA_2_ did not show any appreciable aggregation in the experimental time frame.

## Discussion

Among more than 1000 hemoglobin variants, only a handful of them show structural and functional implications with clinical manifestation. Out of these variants, HbE, the most widespread hemoglobin variant found in the south-east Asia including India, comes out as the one with interesting features. A point mutation in β-globin causes drastic alterations in the surface charge distribution in HbE [4] when compared with HbA and HbA_2_. We have investigated the conformational changes associated with the HbA, HbA_2_ and HbE in terms of their thermal stability under wide temperature range from 4°C-60°C at differents pH. From uv-visible absorption, and synchronous fluorescence spectroscopic measurements, it was revealed that HbE starts disintegrating or melting at a lower temperature (~ 38°C) compared to that of HbA (~ 42°C) and HbA_2_ (~ 45°C) at physiological pH range. These melting temperatures are comparable with the same at high alkaline pH, though a recent study indicated that the melting of HbA starts at much higher temperature (72°C) compared to the value reported here [40]. Though direct *in vitro* measurement of thermal stability of HbE was not performed before, earlier studies on other unstable Hb variants along with HbA and HbA_2_ have shown that thermal stability of HbA_2_ to be the highest among others studied [14,15]. We have also observed that at elevated temperatures, the process of aggregation overtakes the effects of unfolding associated with a sharp decrease in the fluorescence intensity. We have monitored aggregation by 90° light scattering along with dynamic light scattering measurements. Onset of aggregation is seen first in HbE at a significantly lower temperature (~40°C) compared to HbA (~47°C) and HbA_2_ (~50°C) as revealed from DLS measurements. HbE is also found to be more vulnerable to H_2_O_2_ induced oxidation. This observation also has implications to an important clinical observation that patients with HbEβ-thalassemia develop severe anemia [20]. Similar trend was followed in thermal unfolding in acidic pH. However, at pH 4.0, the thermal melting profile did not appear sigmoidal and at a higher temperature, aggregation took place which was also more pronounced in the case of HbE. At pH 2.5, we have observed aggregation from the very beginning resulting in decrease in scattering intensities. 

At highly alkaline pH (pH 11.0 and above) and at elevated temperature, a new fluorophore is formed with characteristic emission at ~ 400 nm in all three hemoglobin variants, characterized to be that of DT. The synchronous fluorescence also showed appearance of DT emission peak. SDS-PAGE studies showed higher yields of high molecular weight bands corresponding to the globin multimers. Since presence of high molecular weight multimers of globin are also associated with generation of DT, it was considered to be of intermolecular nature, though presence of intramolecular DT, in addition to the intermolecular one couldn’t be ruled out. Interestingly, DT formation in hemoglobin has also been shown before [41] where a continuous flux of H_2_O_2_ to oxyhemoglobin gave rise to tyrosine oxidation products like dopamine, dopamine quinine, dihydroxy indole along with DT as the major product. The generation of oxidation products of tryptophan like kynurenine and N-formyl kynurenine was ruled out. Kynurenine, a weak fluorescence emitter, has emission maxima in the region of 490-525nm. N-formyl kynurenine (NFK) has strong emission at around 434nm [42,43]. None of these peaks were observed in the hemoglobin variants. However, though we found out that for HbE, the DT formation takes place at a lower temperature than HbA or HbA_2,_ the densitometric analysis from the SDS-PAGE experiments remained inconclusive to show any difference between the relative quantities of DT formation. 

The average life span of a HbEβ-thalassemic erythrocyte is much shorter than that of normal erythrocyte and oxidative stress has been attributed as a major cause behind this [29]. However, the major hemoglobin variant, HbE has been observed to be thermally less stable and prone to faster aggregation compared to HbA and HbA_2_ in presence of 5 mM H_2_O_2_. The oxidative vulnerability of HbE indicates that it could well be the key factor as hemoglobin oxidation and subsequent damage to membrane proteins and lipids are well documented [23,44-46] and faster oxidative aggregation of HbE could lead to initiation of the destructive processes at a relatively milder oxidative stress for the erythrocytes having HbE. 

## Supporting Information

Figure S1 **The emission spectra of dityrosine as a function of increasing temperature for the three Hb variants (**A**) HbA; (**B**) HbE and (**C**) HbA_2_.** Hb concentrations were kept 1.0 µM for each of them.
(TIF)

Figure S2 **The synchronous fluorescence spectra of tyrosine as a function of increasing temperature at pH 11.5 for the Hb variants (**A**) HbA; (**B**) HbE and (**C**) HbA_2_.** Hb concentrations were kept 1.0 µM for each of them.
(TIF)
