# Supplementary figures and images for: Differential Thermal Stability and Oxidative Vulnerability of the Hemoglobin Variants, HbA2 and HbE
Source: PLoS One. 2013 Nov 14;8(11):e81820. doi: 10.1371/journal.pone.0081820 (PMC3828284; doi:10.1371/journal.pone.0081820)

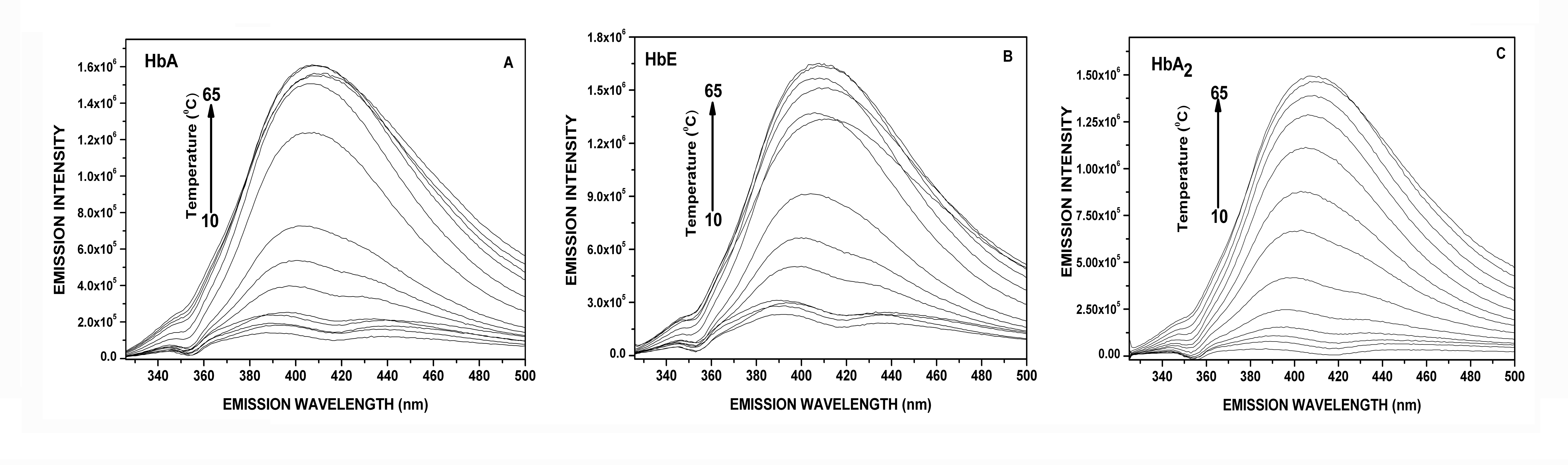

Supplement: Figure S1 — The emission spectra of dityrosine as a function of increasing temperature for the three Hb variants (A) HbA; (B) HbE and (C) HbA2. Hb concentrations were kept 1.0 µM for each of them. (TIF) [file pone.0081820.s001.tif]

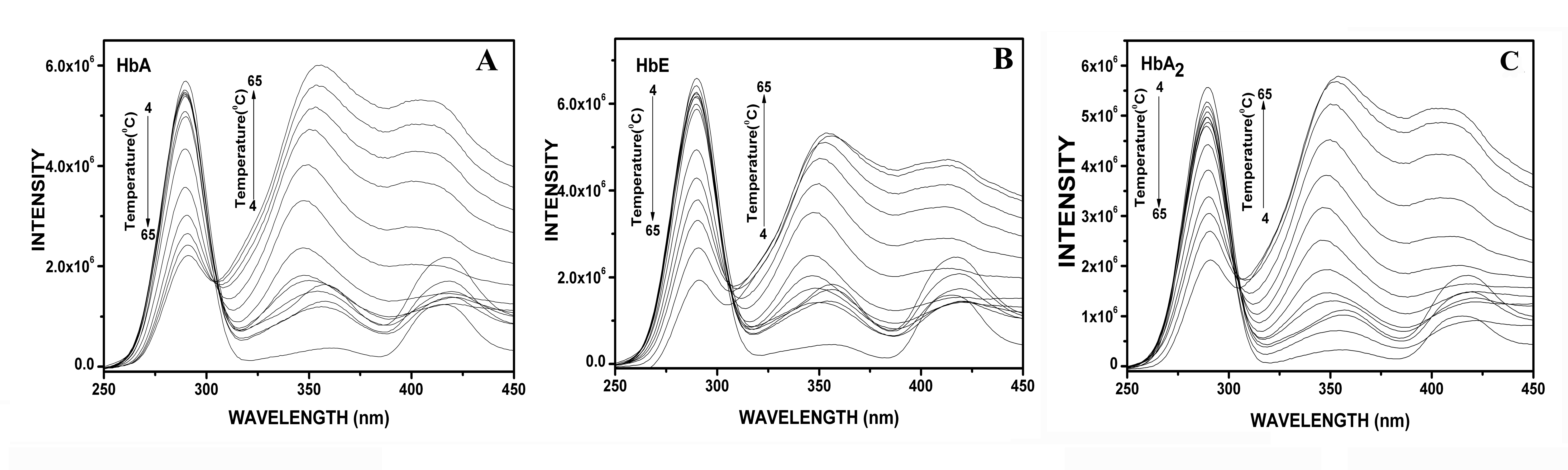

Supplement: Figure S2 — The synchronous fluorescence spectra of tyrosine as a function of increasing temperature at pH 11.5 for the Hb variants (A) HbA; (B) HbE and (C) HbA2. Hb concentrations were kept 1.0 µM for each of them. (TIF) [file pone.0081820.s002.tif]
